# 碳点在抗生素分析检测中的应用

**DOI:** 10.3724/SP.J.1123.2021.04022

**Published:** 2021-08-08

**Authors:** Peijun CHAI, Zhihua SONG, Wanhui LIU, Junping XUE, Shuo WANG, Jinqiu LIU, Jinhua LI

**Affiliations:** 1.烟台大学药学院, 新型制剂与生物技术药物研究山东省高校协同创新中心, 分子药理和药物评价教育部重点实验室, 山东 烟台 264005; 1. School of Pharmacy of Yantai University, Collaborative Innovation Center of Advanced Drug Delivery System and Biotech Drugs in Universities of Shandong, Key Laboratory of Molecular Pharmacology and Drug Evaluation, Ministry of Education, Yantai 264005, China; 2.中国科学院烟台海岸带研究所, 中国科学院海岸带环境过程与生态修复重点实验室, 山东省海岸带环境过程重点实验室, 山东 烟台 264003; 2. Chinese Academy of Sciences (CAS) Key Laboratory of Coastal Environmental Processes and Ecological Remediation, Yantai Institute of Coastal Zone Research (YIC), CAS, Shandong Provincial Key Laboratory of Coastal Environmental Processes, Yantai 264003, China; 3.中国(烟台)知识产权保护中心, 山东 烟台 264003; 3. China (Yantai) Intellectual Property Protection Center, Yantai 264003, China

**Keywords:** 色谱分析, 传感分析, 碳点, 抗生素, 综述, chromatography analysis, sensor analysis, carbon dots (CDs), antibiotics, review

## Abstract

抗生素的过度使用对环境造成了极大破坏,对其进行监测控制刻不容缓。常用的分析检测技术,如高效液相色谱(HPLC)、气相色谱(GC)、高效液相色谱-串联质谱(HPLC-MS/MS)等具有高效快速、重现性好、可自动化操作等优点。但对环境样品中抗生素的检测存在样品前处理过程繁琐、检测灵敏度低、实验成本高等问题。结合现有的检测技术发展新型材料,对提高抗生素的检测灵敏度具有重要意义。碳点(CDs)是一种尺寸介于0~10 nm之间的新型纳米材料,具有小尺寸效应、优异的电学和光学性质、良好的生物相容性等优点,已被广泛应用于环境样品中抗生素的检测。该综述对近5年CDs与传感器、色谱分析技术结合检测抗生素的应用进行了总结,并对其发展前景进行了展望。该文总结了CDs与分子印迹传感器、适配体传感器、电化学发光传感器、荧光传感器及电化学传感器相结合,及其在抗生素检测中的应用;对涉及的比率型传感器、阵列传感器等先进分析方法进行了举例评述;对CDs作为色谱固定相分离抗生素进行了阐述。文献表明,CDs结合传感器检测抗生素可有效提高检测灵敏度,但对复杂环境样品中抗生素的检测还面临着构建高选择性传感器、开发新型材料及数据处理等方面的挑战;目前,CDs作为色谱固定相对抗生素的材料分离,仍处于初步研究阶段,分离机理尚不明确,有待进一步深入研究。总之,CDs在环境样品中抗生素的检测方面仍面临一系列问题,随着人们对CDs的深入研究以及各种分析检测技术的不断发展,CDs将会在抗生素等环境污染物的检测中发挥重要作用。

## Abstract (en)

Antibiotics have been overused in recent years because of their remarkable curative effect, but this has led to considerable environmental pollution. Therefore, the development of approaches aimed at the effective detection and control of the antibiotics is vital for protecting the environment and human health. Many conventional strategies (such as high-performance liquid chromatography (HPLC), gas chromatography (GC), high-performance liquid chromatography-tandem mass spectrometry (HPLC-MS/MS)) are currently in use for the detection of antibiotics. These strategies have aroused a great deal of interest because of their outstanding features of high efficiency and speed, good reproducibility, automation, etc. However, various problems such as tedious sample pretreatment, low detection sensitivity, and high cost must be overcome for the effective detection of antibiotics in environmental samples. Consequently, it is of great significance to improve the detection sensitivity of antibiotics. The development of new materials combined with the existing detection technology has great potential to improve the detection results for antibiotics. Carbon dots (CDs) are a new class of nanomaterials with particle sizes in the range of 0-10 nm. In addition, CDs have desirable properties such as small particle effect, excellent electrical properties, unique optical properties, and good biocompatibility. Hence, they have been widely utilized for the detection of antibiotics in environmental samples. In this review, the application of CDs combined with sensors and chromatographic technology for the detection of antibiotics in the last five years are summarized. The development prospects of CD-based materials and their application to the analysis and detection of antibiotics are presented. In this review, many new sensors (CDs combined with molecularly imprinted polymer sensors, aptamer sensors, electrochemiluminescence sensors, fluorescence sensors, and electrochemical sensors) combined with CD-based materials and their use in the detection of antibiotics are summarized. Furthermore, advanced analysis methods such as ratiometric sensor and array sensor methods are reviewed. The novel analysis methods provide a new direction toward the detection of antibiotics by CDs combined with a sensor. Moreover, CD-based chromatographic stationary phases for the separation of antibiotics are also summarized in this manuscript. It is reported that the detection sensitivity for antibiotics can be greatly improved by the combination of CDs and a sensor. Nevertheless, a literature survey reveals that the detection of antibiotics in complex environmental samples is confronted with numerous challenges, including the fabrication of highly sensitive sensors in combination with CDs. Furthermore, the development of novel high-performance materials is of imperative. In addition, it is important to develop new methods for effective data processing. The separation of antibiotics with CDs as the chromatographic stationary phases is in the preliminary stage, and the separation mechanism remains to be clarified. In conclusion, there are still many problems to be overcome when using CDs as novel materials for the detection of antibiotics in environmental samples. Nowadays, CD-based materials are being intensively studied, and various analytical detection technologies are being rapidly developed. In the future, CD-based materials are expected to play an important role in the detection of antibiotics and other environmental pollutants.

抗生素是由微生物次级代谢产生的或是由人工或半人工合成的一种有机物^[1]^,主要包括:磺胺类、氟喹诺酮类、四环素类等^[2]^。抗生素对微生物的活性有抑制作用^[3]^,可使细菌产生耐药性而减弱治疗效果^[4]^,其滥用会对人体健康产生极大危害^[5]^,且难以通过水净化过程去除^[6]^。世界卫生组织认为,抗生素的耐药性是一场全球的公共危机,需要人们严肃对待^[7]^。因此,建立新的检测方法以检测抗生素的含量,对于管控抗生素污染至关重要^[8]^。常见的检测方法包括:高效液相色谱法^[9]^、液相色谱-串联质谱法^[10]^、毛细管电泳法^[11]^、酶联免疫检测法^[12]^等。为了进一步降低检出限,人们发展了一系列新型材料以辅助上述检测过程,如:分子印迹聚合物(molecularly imprinted polymers, MIPs)^[13,14]^、分析物响应水凝胶^[15]^、有机骨架^[16]^、碳点(carbon dots, CDs)^[17]^等。其中,CDs因优异的物理化学性质而成为研究热点。

CDs又名碳量子点,是一种零基纳米材料,包括碳纳米点(carbon nano dots, CNDs)、石墨烯量子点(graphene quantum dots, GQDs)和聚合物点(polymer dots, PDs)^[18,19]^,其制备分为“自上而下”和“自下而上”两种方法^[20]^。CDs因具有良好的水溶性、生物相容性、独特的光学和电学特性、来源广泛易得等优点^[21,22]^,已被用于检测环境中的金属离子^[23,24]^。最近,人们将CDs与传感分析^[25]^、新型色谱固定相制备等技术结合,以有效检测抗生素。本文对近几年CDs在抗生素分析检测中的应用进行了总结,对所涉及的方法进行了归纳,并对其发展前景进行了展望,以期望新型CDs材料为解决复杂环境样品中的抗生素提供新的契机。

## 1 CDs与传感技术结合检测抗生素

传感器具有高选择性、高灵敏度等性能,能够实现物质的快速检测,被广泛应用于抗生素的测定^[26,27]^。CDs的量子限制和边缘效应^[28,29]^使其能够实现对电子的运输和导电,可有效提高传感器的信噪比^[30,31]^。目前,与CDs结合检测抗生素的传感器主要包括生物传感器、光学传感器、电化学传感器等。

### 1.1 生物传感器

1.1.1 分子印迹传感器

分子印迹技术是一种新型的模板导向技术,该技术合成的MIPs对某一种或某一类分析物具有特异性识别作用^[32]^,可用作传感器中识别元件的替代材料^[33]^。将CDs与MIPs相结合,可有效增强CDs的荧光效应^[34]^,基于MIPs的传感器备受科研工作者青睐^[35]^。

Liu等^[36]^以甘薯皮为原料,以土霉素(oxytetracycline, OTC)为模板分子,制备了MIPs包覆的CDs,将其作为荧光探针用于检测蜂蜜中的OTC,检出限为15.3 ng/mL,且该探针可以重复使用5次以上,大大降低了检测成本。证实了MIPs可以提高CDs对OTC检测的选择性,这种基于MIPs涂层制备CDs的方法对蜂蜜中OTC的含量测定是可行的。在MIPs提高特异选择性的基础上,研究人员为了降低外界环境的干扰,进一步提高检测灵敏度,设计了比率荧光传感器。比率荧光传感器是通过测量两种或两种以上波长的荧光强度比来分析目标物^[37,38]^,其内部的探针具有校正功能,能有效降低检出限。并且该探针的荧光信号可视性强,极大地降低了成本,具有很高的应用价值^[39]^。

Chen等^[40]^以磺胺嘧啶(sulfadiazine, SDZ,由抗生素滥用引起的一种环境污染物)为模板分子,设计了一种比率荧光纳米传感器,用于SDZ的检测,标准样品中SDZ的检出限为0.042 μmol/L。且该传感器可对自来水样品中的SDZ进行检测,回收率可达91.7%~101.2%。随着样品浓度的变化,具有不同颜色的CDs可产生不同程度的荧光淬灭,通过比较加入样品前后的荧光强度变化实现对样品的检测。与单发射的荧光传感器相比,比率荧光传感器可有效避免单荧光传感器引起的误差,减弱检测条件的干扰。Liu等^[41]^构建了分子印迹比率传感器(MIPs@rCDs/bCDs@SiO_2_),在0~50 nmol/L的线性范围内,标准样品中四环素(tetracycline, TC)的检出限为1.19 nmol/L,且已被证实对来自河水、自来水中等实际样中的TC能实现准确定量。虽然这是一种高选择性的体系,但CDs作为荧光淬灭的信号输出,易受到仪器和环境的影响,重现性有待提高。比率传感器除对环境等复杂样品有很好的检测外,还可以对牛奶等含有痕量抗生素的样品实现高灵敏检测。Jalili等^[33]^报道了双荧光团比率荧光传感器(见图1),该传感器的响应时间约为5 min,为非印迹传感器响应时长的1/8,其对牛奶中青霉素(penicillin, PNG)的检出限为0.34 nmol/L。

**图1:**
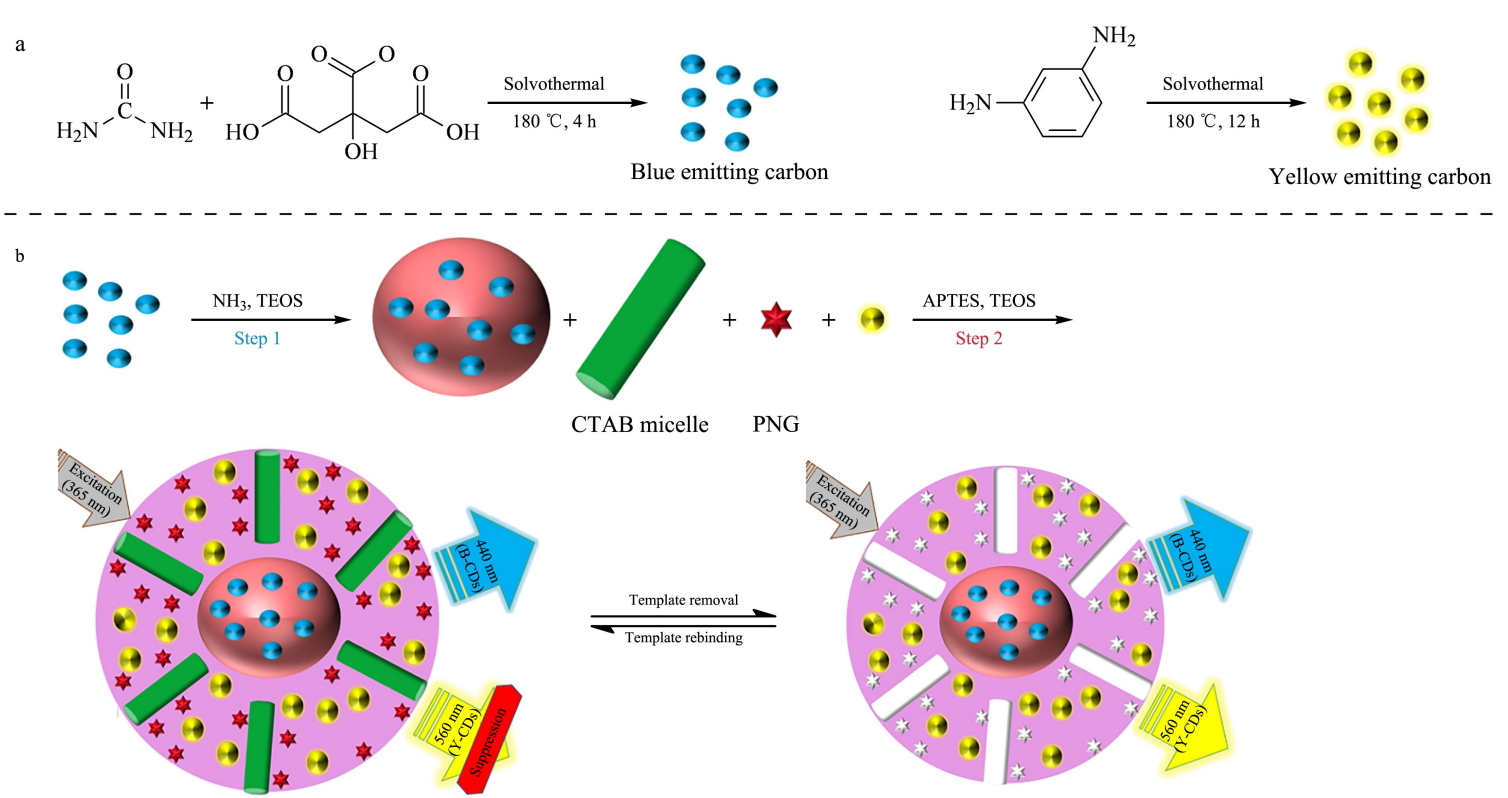
(a)碳点的制备和(b)比率传感器的制备及青霉素传感机理示意图^[33]^

该方法具有快速、灵敏度高的优点^[42]^。该课题组^[43]^还将该类传感器用于标准样品中氯霉素(chloramphenicol, CAP)的检测,线性响应范围为0.1~3 μg/L,检出限为0.035 μg/L。Sahebi等^[44]^采用超高效液相色谱-串联质谱法(UHPLC-MS/MS)对牛奶中的PNG等5种抗生素进行分析,其检出限为0.03~0.20 μg/kg;此外,崔敬鑫等^[45]^采用UHPLC-MS/MS对水样中氯霉素类、四环素类等15种抗生素同时进行测定,其检出限为2.1~22.0 ng/L。由此可见,基于MIPs的比率传感器,其检出限与UHPLC-MS/MS的检出限可达到相同的数量级,且比率传感器无需昂贵的生物识别分子或复杂的传感系统,具有强大的可视化效果,易于携带,可用于现场检测。

1.1.2 适配体传感器

适配体是由20~60个核苷酸组成的单链DNA或RNA分子^[46]^,类似于抗体,适配体具有易于标记、合成简单以及与目标物质结合能力好的优点,被广泛用作传感器的识别元件^[47,48]^。

Wang等^[49]^用枸橼酸包被的金纳米粒子(AuNPs)作为吸收剂,构建了一种新型无标记的适配体传感器来检测食品中的卡那霉素。该传感器基于内滤效应对标准样品中卡那霉素检测的线性范围为0.04~0.24 μmol/L,检出限为18 nmol/L,对牛奶样品中卡那霉素检测时,回收率可达到98%。基于内滤效应的检测方法不需要CDs与AuNPs相连接,仅需要AuNPs的吸收光谱与CDs的荧光激发光谱有重叠即可,有效简化了实验步骤,实用性强;且用枸橼酸修饰的AuNPs提高了荧光淬灭效率,结合适配体的特异性识别,能达到满意的检测效果。但这种基于原子吸收光谱分析的适配体传感器存在荧光团使用寿命短、易受背景荧光及环境影响的问题。Roushani等^[50]^开发了基于AuNPs和巯基石墨烯量子点(GQD-SH)的传感器,用于检测牛奶和血清中的链霉素(streptomycin, STR)。在此基础上,该课题组^[51]^又提出了基于胺基和巯基功能化的GQDs-*N-S*的新型核酸适体传感器,将银纳米粒子(AgNPs)包覆在玻碳电极(GCE)上以检测标准样品中的STR,其检出限为0.0033 pg/mL,对血清中的STR检测时,回收率高达99.03%。此外,Roushani等^[52]^还通过将硫脲包覆的ZnS量子点和AuNPs包覆于GCE表面,建立了一种硫醇适配体修饰的传感系统,用以检测标准样品中的STR,其检出限为0.35 fg/mL,极大提高了检测灵敏度,并证实可对实际样品中血清等生物样品中的STR进行检测。这种基于电化学阻抗谱方法制备的传感器具有较高的选择性,良好的重现性及稳定性,对解决环境等复杂实际样品中抗生素的检测难题具有潜在应用价值,但构建该类传感器过程复杂,成本高,因此发展新型材料对该领域十分必要。

MIPs具有与模板分子在形状、大小、官能团互补且完全匹配的结合位点,其独特的识别能力以及极高的物理稳定性使其成为生物传感器识别元件的不二选择;相比于传统的识别元件(抗体等),适配体易于合成,稳定性强,价格便宜,且适配体序列具有很强的灵活性,易于标记和修饰,被认为是最有希望的替代元件。将CDs与MIPs及适配体相结合,有望提高传感器的检测灵敏度及检测结果的准确性。相比于适配体传感器,MIPs传感器具有更强的机械稳定性,但其与目标物的结合能力稍差^[53,54]^,在实际应用中可根据目标分子的特点进行选择。例如,Geng等^[55]^在以卡那霉素为模板分子、硒化镉(CdSe)量子点为载体、甲基丙烯酸为功能单体的基础上,加入了经巯基修饰的适配体作为另一种功能单体,使合成的传感器实现了对卡那霉素的高灵敏检测。在0.05~10.0 μg/mL范围内,标准样品中卡那霉素的检出限为0.013 μg/mL,在对来自湖水、自来水中的卡那霉素进行检测时,均可得到满意的结果。将适配体和MIPs的印迹空穴相结合,能实现对分析物的双重识别,提高检测灵敏度;此外,量子点表面的活性基团有利于在其表面形成MIPs层。将MIPs及适配体与传感器相结合,使检测效果更加显著,这种策略是检测复杂环境样品中抗生素的有效方法。另外,Roushani等^[54]^将适配体传感技术与分子印迹技术结合,增强了传感性能,标准样品中CAP的检出限为0.3 pmol/L,对实际样品牛奶中CAP的回收率可达103%。

### 1.2 光学传感器

光学传感器因具有操作简单、稳定性好等优势,成为抗生素检测方法的研究热点^[56]^。按照检测方法的差异,光学传感器可分为化学发光法、荧光法等,这两种方法在抗生素检测中最常用^[57]^。基于量子点的光学传感器因具有宽吸收光谱和窄发射光谱,备受研究者关注。

1.2.1 电化学发光传感器

电化学发光(electrochemiluminescence, ECL)是一个将电化学和光谱学结合的技术,当电极发生电子转移反应达到激发态时,电极上生成的物质就会发光^[58]^。这使得ECL不需要借助外部光源,具有快速响应、背景噪音低的优点^[59,60]^。CDs可有效提高电极的电化学活性^[61]^,将CDs引入ECL传感器,大大提高了检测灵敏度。

He等^[62]^将硫化镉量子点吸附到氧化石墨烯纳米材料表面,然后将其修饰在玻碳电极表面,在1.0×10^-12^~1.0×10^-7^mol/L的线性范围内,标准样品中CAP的检出限为0.5 pmol/L,对牛奶中CAP的回收率为97%~103%。Hu等^[63]^利用共反应物和发光基团之间的电化学反应可产生ECL信号的原理,采用尿素和EDTA制备了CDs共反应物,以Ru(bpy ${)}_{3}^{2+}$ 为发光团,产生双ECL信号,用于检测标准样品中的TC,可避免单信号检测引起的误差。在1.0 nmol/L~0.1 mmol/L范围内,信号比值与浓度的对数呈线性关系,其检出限为0.47 nmol/L。该方法对牛奶中TC检测时,回收率为88.9%~104.6%。ECL能够将电信号转化为光信号^[53,64]^,而CDs作为新型的ECL发光体,可提高检测灵敏度,拓宽线性范围。CDs为创建一种高效的ECL传感器提供了可能,但CDs结合ECL传感器在检测抗生素方面仍具有很大的发展空间,未来可在CDs的基础上进行杂原子掺杂、表面修饰以及优化ECL电极方面继续研究,以弥补复杂环境样品中抗生素检测的缺陷。

1.2.2 荧光传感器

荧光检测具有响应速度快、灵敏度高等优点,常用来检测复杂基质中的抗生素。CDs因具有独特的荧光特性、良好的生物相容性^[65]^,常被作为荧光探针来分析目标物,是荧光检测的优良材料。Yu等^[66]^以赖氨酸为原料,采用微波法合成了CDs,并将其用于抗生素类化合物的检测。TC、多西环素(doxycycline, DOX)等7种抗生素在290 nm和365 nm的光激发下,对CDs表现出不同的荧光猝灭现象。此外,将Al^3+^离子与CDs进行络合后,不仅可改变其荧光强度^[67]^,还会使7种抗生素的发射峰有所差异,由此,可根据峰位置和荧光强度的变化对7种抗生素进行定性分析(见图2),其对各种标准样品中抗生素的检出限均低于50 nmol/L。

**图2:**
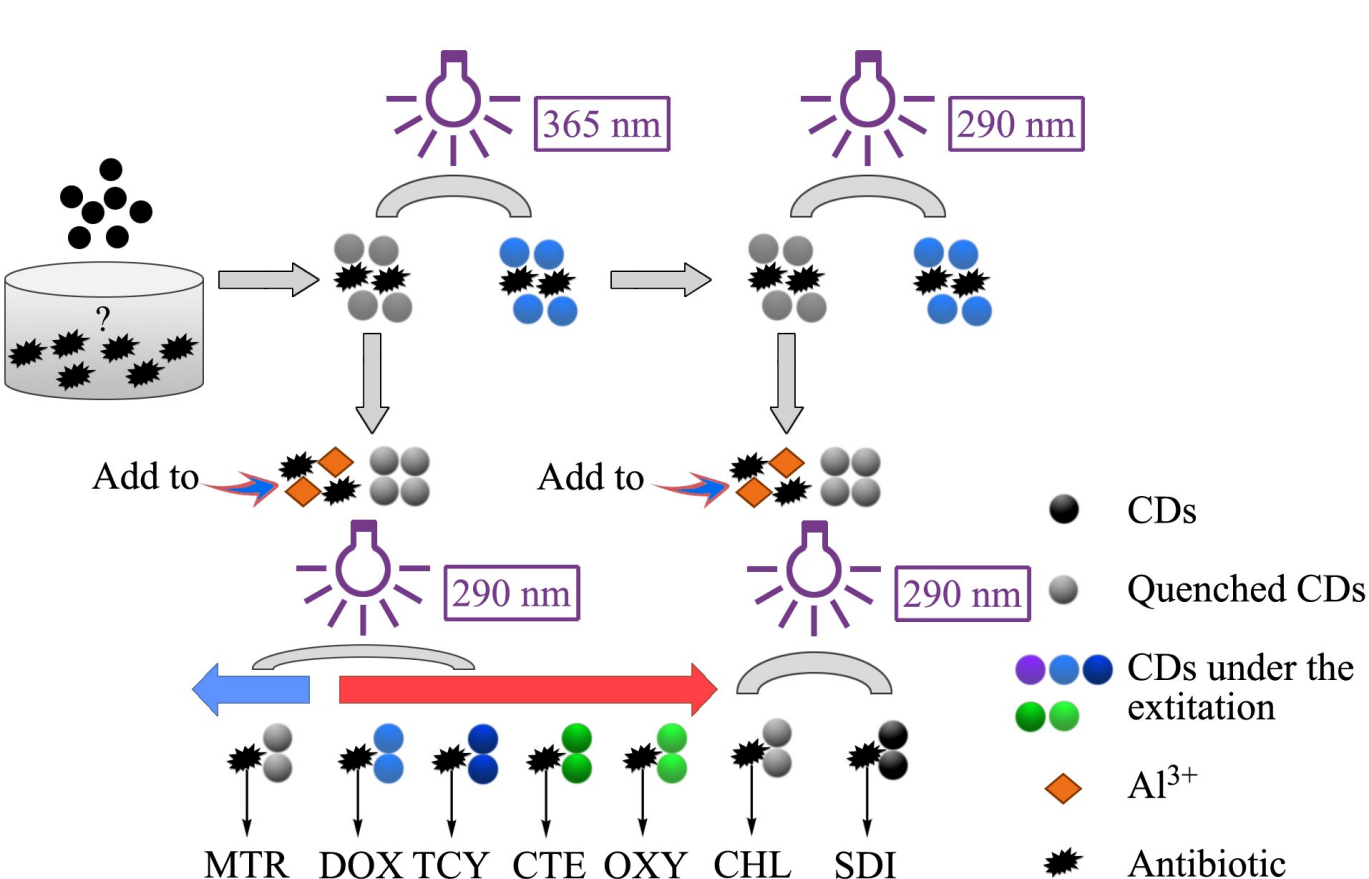
7种抗生素的两步检测流程图^[66]^

该方法证明,CDs是基于内滤效应以及静态淬灭效应来检测抗生素,为人们研究利用CDs检测抗生素提供了理论依据,开辟了新思路。为了增强CDs的荧光性能,研究人员^[68]^通过其他原子的掺杂来提高检测能力。Chen等^[69]^开发了一种N、B、F共掺杂碳点(N,B,F-CDs),并将其用于标准样品中磺胺噻唑(sulfathiazole, STZ)的检测,其检出限为5.5 ng/mL。将该传感器对来自土壤、河水中的STZ进行检测,其回收率为96.7%~101.0%,该制备方法简单、灵敏,环境友好,对环境样品中STZ的检测具有巨大的应用价值,但该方法需要对样品进行繁杂的前处理,无法直接对实际样品进行现场检测。除了基于内滤效应和静态淬灭效应检测抗生素外,Fu等^[70]^首次提出了基于共振能量转移(Förster resonance energy transfer, FRET)机理的CDs荧光探针检测标准样品中OTC,其检出限为0.41 μmol/L,并对河水、自来水及矿泉水中的OTC进行测定,其回收率为95.0%~105.0%,该方法无需引入其他荧光基团,改善了探针的重复性,为实现抗生素的高灵敏检测提供了一种新思路。基于CDs的荧光探针的优点包括:所用试剂价格便宜、选择性高、灵敏度高。CDs优异的荧光特质提高了荧光传感器的传感性能,但对抗生素的分离能力有限,因此发展选择性强的CDs材料以及与各种新型材料、传感技术、检测方法相结合对于环境中抗生素的检测具有重要意义。

为了使检测结果更加直观、准确,人们开发了比色以及比率传感的方法。Miao等^[71]^以烟草为原料合成CDs,将其作为荧光探针,用于3种抗生素标准样品TC、金霉素(aureomycin, CTC)、OTC的检测,检出限分别为5.18、6.06、14 nmol/L。此外,Miao等^[71]^将含有CDs的液滴浸润待测物,再采用紫外灯照射,根据颜色变化对3种抗生素进行检测。该方法具有简单、易操作、结果明显的特点,可作为荧光探针用于环境污染物的痕量检测。Hu等^[72]^通过将蓝色CDs与单磷酸胞苷(CMP)/铕配位聚合物纳米粒子结合,设计了一种比率荧光传感器的探针BCDs-Eu/CMP-cit,并将其用于TC的检测。该方法具有双信号响应性,在检测牛奶等实际样品时,所得回收率、相对标准偏差等参数与高效液相色谱法的检测结果相当。同时,将含有BCDs-Eu/CMP-cit探针的滤纸条用来检测牛奶、蜂蜜、牛肉中的TC,当样品中TC浓度高于0.05 μmol/L时,用肉眼可直接观察到滤纸条的颜色随TC浓度改变而发生变化,该方法具有巨大的实际应用潜力。在荧光检测的基础上,借助比色和比率的研究方法,使得检测结果更加清晰、准确。CDs与不同的抗生素结合可在不同的波长下呈现出不同的颜色,比色法的实验现象明显、操作简单、无需借助其他复杂仪器就可得到最直观的结果,但其抗干扰能力较弱,易受环境影响;而比率荧光探针通过两个荧光发射强度的比值来检测分析物,克服了单个荧光探针产生的误差,有效提高了准确度。两种策略在保障低检出限的基础上,对检测结果的直观性、准确性有了进一步的提高,扩宽了荧光传感器的应用。

在比率传感器的基础上,人们又提出了阵列型传感器,该传感器可同时对多种成分进行检测,且无需极高的特异性受体^[73]^,已被广泛应用于多种目标物的测定^[74,75]^。Mao等^[76]^设计了一种交叉反应传感器阵列来检测OTC、TC等8种标准样品抗生素,用异亮氨酸(isoleucine, Iso)等4种氨基酸作为CDs的非特异性受体,该受体可与目标分析物相互作用,采用凝胶生物成像系统对其荧光强度的变化进行检测,产生特定的“指纹”图谱。当加入4类(8种)抗生素时,被修饰的CDs荧光强度会发生变化,可对8种抗生素进行测定。该传感器的识别示意图、荧光响应、线性判别分析(linear discrimination analysis, LDA)分析图见图3。

**图3:**
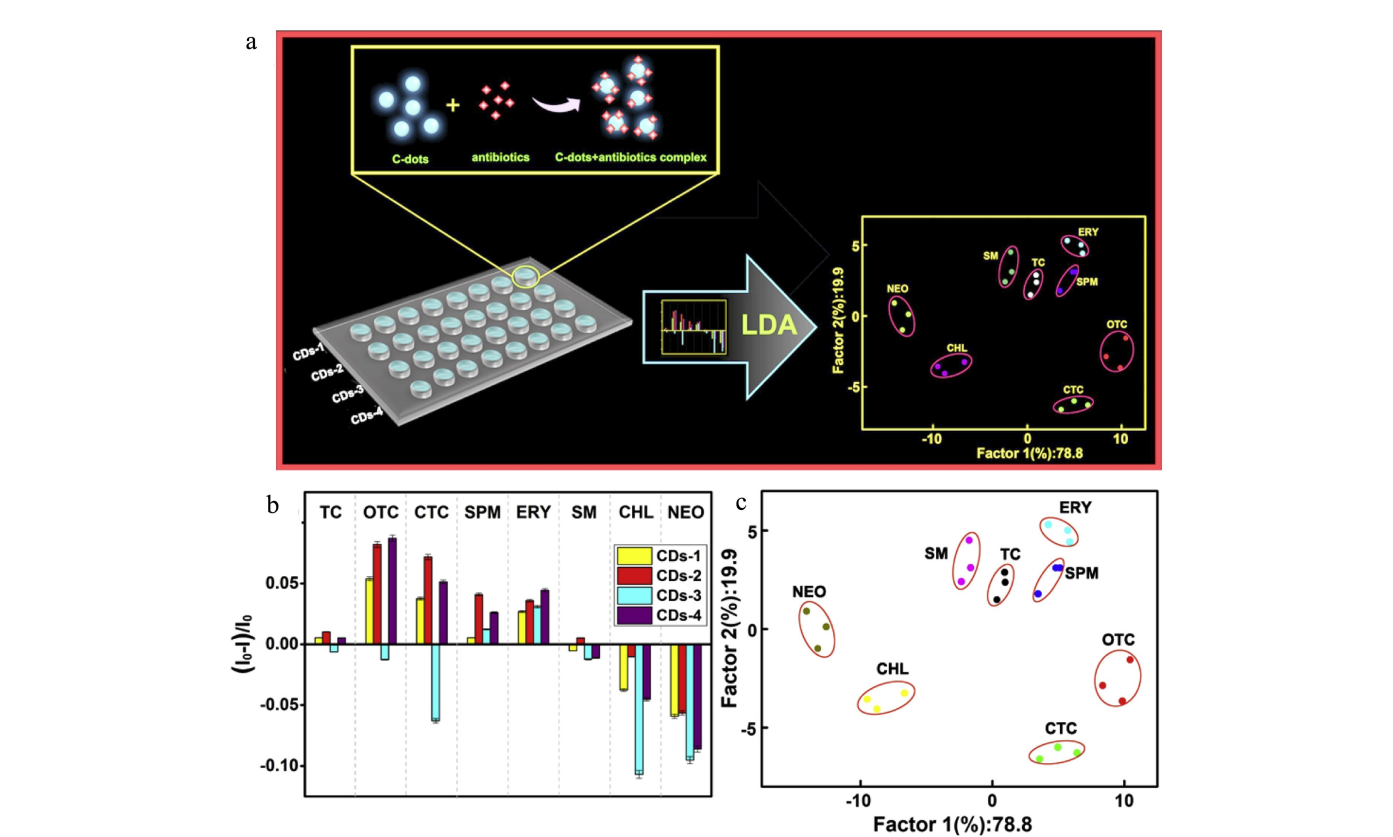
抗生素识别原理及结果分析图^[76]^

该工作可以在短时间(几秒钟)内得到所有物质的荧光变化谱图,可对人尿液等复杂介质中8种抗生素进行检测。Long等^[77]^设计了一种无标记的四通道荧光阵列传感器,用以检测TC等4种抗生素标准样品,以绿、蓝色CDs以及它们的混合物作为传感元件,能对浓度为1 μmol/L的4种抗生素实现准确鉴别,且对牛奶中的TCs具有很好的响应。该方法不引入任何金属离子和有害物质,也无需复杂的装置,具有环境友好、操作简单的优点。Xu等^[78]^开发了双通道荧光传感器阵列,可有效区分浓度为10 μmol/L的4种标准样品抗生素(TC、OTC、美他环素(metacycline, MTC)、DOX),对河水和牛奶等实际样品中的TCs进行鉴定,准确率高达100%。传感器阵列对复杂基质中的多种抗生素检测具有分析时间短、灵敏度高、干扰小、准确度高等一系列优势,且对结构、性质相似的物质也能进行良好的区分检测。结合CDs的阵列传感器虽比其他检测方法提供更多的信息,但存在数据处理和分析方法上的问题,仍需继续改进。如常用的主成分分析法(principle component analysis, PCA),常由于忽略内部信息,而造成不必要的分类;LDA虽能解决这一问题,但在分析处理非线性数据时存在弊端;作为能处理线性和非线性数据的支持向量机(support vector machine, SVM)已被证实可以用于传感阵列检测抗生素,但其也存在分类上的问题。因此,利用阵列传感器检测抗生素时,在阵列传感器构建以及数据处理方面仍需进一步改进,对提高抗生素检测灵敏度有重大意义。

### 1.3 电化学传感器

电化学方法因具有灵敏度高、便携、经济、环境友好的特点,被应用于环境污染物的检测^[79,80]^。采用CDs修饰电极可增大其表面积,使其捕获更多的分析物,CDs与电化学结合也成为该领域的新兴发展趋势^[81]^。

Huang等^[82]^采用电聚合法合成聚邻氨基苯酚(PoAP)/GQD膜包覆的GCE,以提高传感器对左氧氟沙星(levofloxacin, LV)的检测能力。该仪器对标准样品中LV的检出限为10 nmol/L,信噪比为3,与其他电化学材料(如MIP/G-Au/GCE)相比,具有更宽的检测范围,更低的检出限,对牛奶中的LV测定时,其回收率可达96.0%~101.0%。GQD具有很好的水溶性,可通过静电力与LV结合,且与PoAP之间的*π*-*π*堆积作用使得GQD能吸附在GCE上,增强其导电性。为了增强GQD在GCE上的稳定性,研究者还设计使用了其他的材料。如Gondim等^[83]^采用GQD@Nafion(Nafion即全氟磺酸隔膜,可被用作阳离子传导膜和电子屏障,用于增强电极表面纳米粒子的稳定性^[84]^)对玻璃碳电极进行修饰,并用于测定牛奶中的磺胺类药物,结果令人满意。CDs具有良好的导电性,使得经CDs修饰后的电极具有更高的选择性。此外,人们还通过CDs与其他纳米材料相结合的手段,进一步降低抗生素的检出限。Muthusankar等^[85]^合成了Co_3_O_4_包覆氮掺杂碳点负载于多壁碳纳米管表面的复合材料(N-CQD@Co_3_O_4_/MWCNTs),以测定呋喃妥因(NF)。将该复合材料涂覆在GCE表面(N-CQD@Co_3_O_4_/MWCNT/GCE)使得电极和电解质界面接触充分,利于电荷转移,标准样品中NF的检出限为0.044 μmol/L,有效提高了检测灵敏度。该体系中载体多壁碳纳米管具有较大的表面积,使得电子传输更迅速;N-CQDs与MWCNTs之间的疏水作用,增强了复合材料的稳定性及电化学活性。

CDs能提高电化学传感器的传感性能,但CDs不能直接吸附在GCE上,这不利于电化学传感器检测结果的重现性,且会缩短传感器的使用寿命。虽然,目前已有很多复合材料、载体辅助CDs修饰GCE,但仍需开拓更多的材料,协助CDs检测更多种类的抗生素。此外,发展尺寸可控CDs的制备方法,并探索CDs对目标分析物的响应机理,将有利于进一步改善CDs对抗生素检测结果的可靠性。

## 2 CDs与色谱技术结合用于抗生素的检测

色谱技术的发展主要依靠色谱固定相的制备技术和新的检测手段,固定相作为色谱柱的核心^[86]^,直接影响化合物的分离,为了有效改善抗生素类化合物分离结果的准确性,人们发展了一系列新型色谱固定相。其中,CDs因其表面具有亲疏水性基团、尺寸小、在硅胶表面分布均匀等特性,可与多孔硅胶结合用作色谱固定相,用于抗生素类化合物的有效分离。

Yuan等^[87]^设计并合成了一种葡萄糖衍生的CDs,用于修饰多孔硅胶微球(Sil-Glc-CDs),可对氧氟沙星(ofloxacin, OFL)、罗红霉素等6种抗生素标准样品实现完全分离,且对罗红霉素的分离柱效高达63000 N/m,与非掺杂CDs的色谱柱(Sil-Glc柱)相比,具有更好的色谱分离性能。CDs作为固定相的修饰材料,不仅具有丰富的反应位点,而且能保障填料的均匀性^[19]^。在此基础上,该团队又制备了氮掺杂CDs修饰多孔硅胶色谱固定相^[88]^,实现了罗红霉素等7种标准样品抗生素的分离,与商业柱XBridge HILIC、Globalsil^TM^ Amino以及Sil-Glc-CDs相比,具有更好的分离性能。该固定相可在9 min内实现罗红霉素胶囊中罗红霉素的有效分离,对罗红霉素测定的理论塔板数为50100 N/m。此外,这两种固定相对人参皂苷、氨基酸等也具有很好的分离效果。Wu等^[89]^开发了一种新型两亲性CDs,与多孔硅胶结合制备的固定相,同时具有亲水性和疏水性,对标准样品中的抗生素、核苷以及多环芳烃等物质具有良好的分离效果,该类两亲性CDs修饰硅胶固定相在混合分离模式下具有良好的发展前景。此外,在色谱固定相中引入CDs能有效改善多孔硅胶固定相的峰拖尾现象,有效提高分离柱效。虽然,CDs是一种优异的色谱材料,但基于CDs合成材料直接用于环境样品中抗生素分离分析相关工作的报道较少^[90]^,发展尺寸可控、多功能化的CDs对解决环境样品中抗生素的分析检测问题具有重要意义。

此外,碳点以及其他碳材料也被用作吸附材料,分离检测抗生素。如Yang等^[91]^首次制备了一种基于零维N、S共掺杂碳点、二维金属有机骨架(metal organic frameworks, MOF)以及三维锆(Zr)-MOF的智能吸附剂UiO-67/NSCN,用于水中TC的检测,在0.08~20.0 mg/L范围内,TC的检出限为0.063 mg/L,该吸附剂对TC具有很好的识别能力。当该吸附材料转化为二维纳米材料时,对TC的吸附量可达到427.35 mg/g;当转化为三维材料时,可实现水中TC的去除,且UiO-67/NSCN已被证明是一种无毒安全、智能化材料,该方法有效改善了碳点的分散性能,使其可作为吸附剂分离抗生素。Peng等^[92]^利用分子印迹聚合物的高选择性以及碳纳米管的强吸附能力,对传统的搅拌萃取进行了改造,用于测定水样中痕量头孢克洛和头孢氨苄,其富集系数分别为45.5和45.2,检出限分别为3.5 ng/mL和3.0 ng/mL,磁性碳纳米管的加入,使得吸附、洗涤、洗脱这些步骤一步就可以完成。CDs作为一种新型纳米材料,虽具有较大的表面积和吸附能力,但目前合成的CDs具有大量的活性基团(如-COOH等)以及很强的分散能力^[93]^,这使得CDs作为吸附材料在抗生素样品前处理中的应用受限,因此需要发展不同类型的CDs以及合成方法来解决这一问题。

此外,CDs良好的生物相容性以及分散性,使得CDs可与抗生素结合,在色谱仪器检测下实现更低的检出限。Lahouidak等^[94]^用水热蚀刻法制备了CDs,将CDs加入抗生素的水溶液中,采用毛细管电泳分离,结合荧光检测,对牛奶中的OFL进行分析,检出限和定量限分别为10.7 ng/mL和35.5 ng/mL。此外,上述体系还可用于7种标准氟喹诺酮类抗生素样品的分析检测,与HPLC等方法相比,具有成本低、消耗有机溶剂少等优点。采用GQD使毛细管电泳对目标物的检出限达到μg/L级,比固相萃取-毛细管电泳法测得的检出限低40倍^[95]^,且分析速度更快。另外,Taranova等^[96]^基于不同颜色的水溶性量子点(quantum dots, QDs)的标记作用,建立了能够检测复杂基质中抗生素的免疫层析法,即“交通灯”法(见图4)。该工作对标准样品中OFL、CAP、STR的检出限分别为0.3、0.12和0.2 ng/mL,比酶联免疫法测得3种抗生素的检出限低40~300倍^[97,98,99]^,对牛奶样品中OFL进行检测时,检测时间仅为10 min,是ELISA法的1/18。随着样品浓度的升高,相应测试区的颜色强度降低为零,该方法检测范围宽、重复性好、灵敏度高,分析物可回收且仪器检测的误差小(不大于8%),具有很强的实用价值。

**图4:**
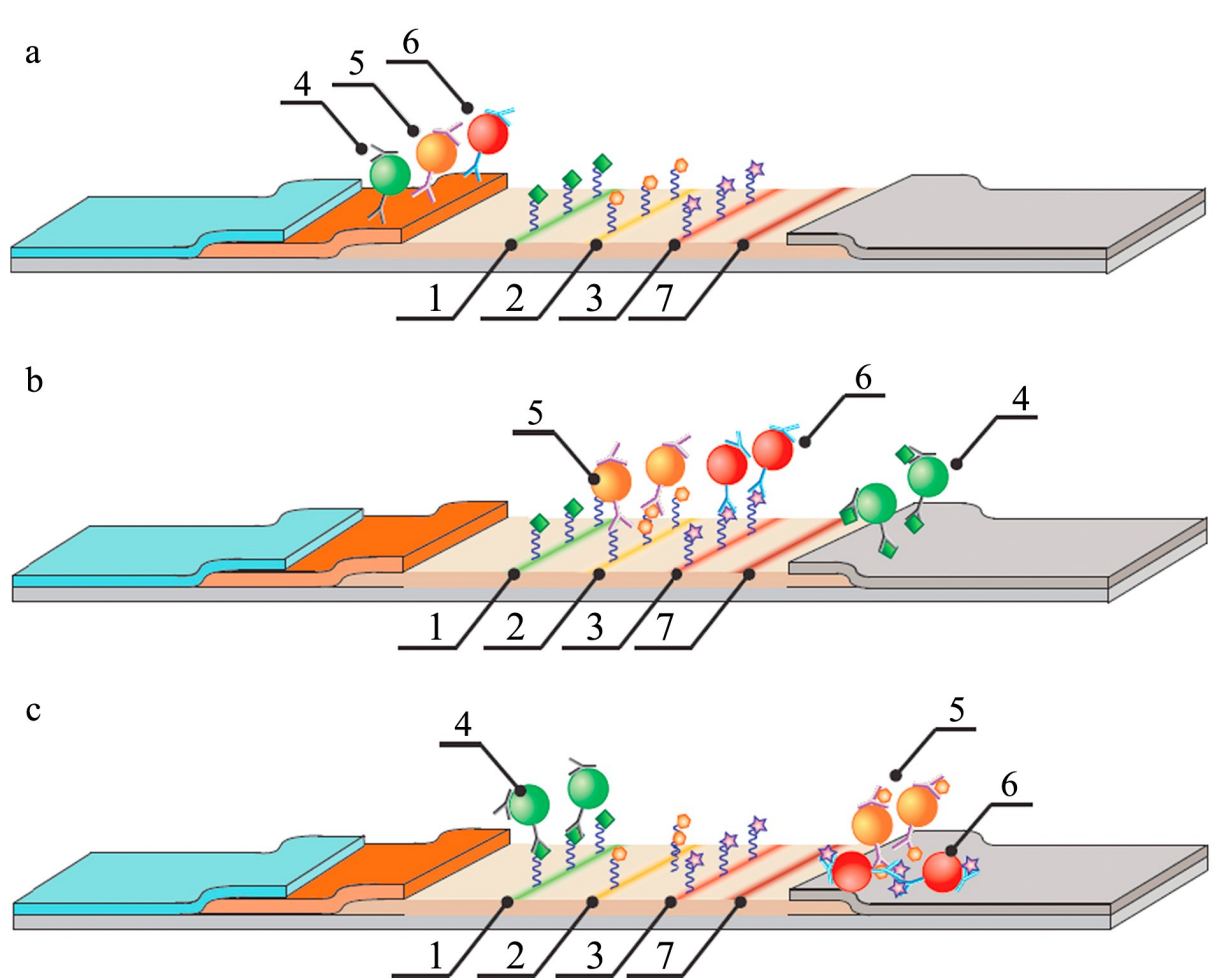
“交通灯”免疫层析法检测抗生素的原理图^[96]^

CDs具有稳定的碳核和丰富的表面基团,可作为分离材料用于色谱分离。利用CDs合成的固定相具有更强的选择性和识别性,已引起研究者的关注。传统的色谱分析(高效液相色谱法、气相色谱法等)与之相比,虽具有高灵敏度和准确性,但存在样品前处理过程复杂、耗时、消耗有机溶剂多等问题^[100]^。CDs与色谱技术结合可在一定程度上简化样品前处理过程、提高检测灵敏度以及节约成本。但目前基于CDs作为色谱分离材料的机理还不清楚,有待进一步研究^[19]^。

## 3 总结与展望

本文针对CDs在抗生素检测中的应用,对近5年发表的相关文献进行了总结归纳。包括以下几点:(1)重点介绍了CDs在传感器中的应用,CDs独特的光学、电学等性质有效提高了传感器检测灵敏度,使其成为传感器中最具潜力的替代材料。如今,基于CDs的生物传感器、光学传感器、电化学传感器被广泛应用于抗生素的检测,实现了环境及食品基质中抗生素的有效检测。(2)CDs的小尺寸效应及两亲特性,使其易与色谱技术结合用于抗生素的检测,但目前相关研究仍然较少,这可能与抗生素的检测基质复杂、可用于抗生素前处理的CDs种类有限,以及CDs作为色谱分离材料的机理并不完全清楚有关。(3)目前,基于CDs材料检测抗生素的基质比较单一,对湖水、土壤等复杂环境样品中抗生素的分析检测还相对较少,且由于CDs的尺寸、功能在合成过程中易受实验温度、时间以及实验人员操作水平的影响,所以对于一些小尺寸、功能特殊的CDs合成还处在实验室阶段。在未来,发展新型的CDs制备技术及材料掺杂技术,合成性能优异的CDs,并深入研究基于CDs材料的作用机理,对解决环境样品中抗生素的分析检测难题具有重要意义。
